# Whole Body Vibration Improves Brain and Musculoskeletal Health by Modulating the Expression of Tissue-Specific Markers: FNDC5 as a Key Regulator of Vibration Adaptations

**DOI:** 10.3390/ijms231810388

**Published:** 2022-09-08

**Authors:** Ida Cariati, Roberto Bonanni, Gabriele Pallone, Cristian Romagnoli, Anna Maria Rinaldi, Giuseppe Annino, Giovanna D’Arcangelo, Virginia Tancredi

**Affiliations:** 1 Department of Clinical Sciences and Translational Medicine, “Tor Vergata” University of Rome, Via Montpellier 1, 00133 Rome, Italy; 2 Department of Systems Medicine, “Tor Vergata” University of Rome, Via Montpellier 1, 00133 Rome, Italy; 3 Department of Industrial Engineering, “Tor Vergata” University of Rome, Via Montpellier 1, 00133 Rome, Italy; 4 Centre of Space Bio-Medicine, “Tor Vergata” University of Rome, Via Montpellier 1, 00133 Rome, Italy

**Keywords:** whole body vibration, synaptic plasticity, hippocampus, cerebellum, musculoskeletal system, FNDC5, BDNF, myostatin, COL-1, vibratory training

## Abstract

Whole body vibration (WBV) is well known to exert beneficial effects on multiple tissues, improving synaptic transmission, muscle mass, bone quality, and reducing anxiety and depressive behavior. However, the underlying molecular mechanisms are not yet fully understood, and organs and tissues may respond differently to the vibratory stimulus depending on multiple factors. Therefore, we investigated the WBV effects on the brain and musculoskeletal tissue of 4-month-old young mice, evaluating synaptic plasticity by electrophysiological recordings and tissue organization by histology and histomorphometric analysis. Specifically, WBV protocols were characterized by the same vibration frequency (45 Hz), but different in vibration exposure time (five series of 3 min for the B protocol and three series of 2 min and 30 s for the C protocol) and recovery time between two vibration sessions (1 min for the B protocol and 2 min and 30 s for the C protocol). In addition, immunohistochemistry was conducted to evaluate the expression of fibronectin type III domain-containing protein 5 (FNDC5), as well as that of tissue-specific markers, such as brain-derived neurotrophic factor (BDNF) in brain, myostatin in muscle and collagen I (COL-1) in bone. Our results suggest that the WBV effects depend closely on the type of protocol used and support the hypothesis that different organs or tissues have different susceptibility to vibration. Further studies will be needed to deepen our knowledge of physiological adaptations to vibration and develop customized WBV protocols to improve and preserve cognitive and motor functions.

## 1. Introduction

Whole-body vibration (WBV) has long been well known to be a powerful stimulant for the entire organism [1,2], with effects on different systems and tissues that can be extremely variable, being highly dependent on the vibration frequency, the number of vibration sessions and the recovery time between two consecutive sessions [3,4,5,6]. Therefore, the development of vibratory training protocols designed on individual needs could be a valid strategy to counteract chronic and/or degenerative pathologies in immobilized patients.

Several studies have reported a powerful effect of WBV on the nervous system, including the enhancement of hippocampal synaptic plasticity, suggesting its potential role in preventing and/or counteracting age-related cognitive decline [7,8]. In this context, we recently studied changes in synaptic plasticity by means of hippocampal electrophysiological recordings in a group of 4-month-old mice exposed to different WBV protocols [9]. Surprisingly, the protocol characterized by a lower vibration frequency and longer recovery time positively modulated hippocampal synaptic plasticity, suggesting an effect of the WBV protocol on higher cognitive processes of learning and memory. In contrast, the other vibratory training protocols used, which differed in vibration frequency, vibration exposure time, and recovery time, induced epileptic tendency development, highlighting the appearance of brain damage induced by the over-stressful WBV training [9]. In agreement, Raval et al. demonstrated that exposure to a low-frequency WBV protocol for 30 days significantly improves neurological and motor skills in female rats, as well as preventing post-ischemic cognitive decline, probably by a multifactorial mechanism that reduces post-stroke inflammation and frailty [10].

WBV effects have also been extensively documented at the musculoskeletal level, suggesting among the main benefits are an increase in bone mineral density and muscle mass and strength, as well as improved motor performance and general health [11,12]. Furthermore, WBV is currently used as a preventive tool for several diseases, such as osteoporosis, sarcopenia, and chronic low back pain, as well as for improving muscle function and joint stability [13,14,15]. In this context, we recently investigated the effects of WBV on skeletal muscle in a group of 12-month-old mice by light and electron microscopy, observing significant variations depending on the vibratory training protocol used [16]. Again, the most satisfactory results were observed in the group of animals subjected to the vibratory protocol characterized by the lowest frequency, shorter vibration exposure time, and longer recovery period between two consecutive sessions. In contrast, a more stressful WBV protocol was correlated with a higher percentage of atrophic fibers, altered sarcomeric structures, and abundant interfibrillar fibrosis [16]. Similarly, Matsumoto and colleagues studied the effects of low-intensity WBV in 13-week-old mice undergoing tibial perforation, observing an improvement in bone healing induced by an increase in vascular growth [17].

Although the molecular mechanisms by which WBV exerts its action on the entire organism have not yet been elucidated, exercise is known to modulate the production of a wide variety of molecules that can act with paracrine or endocrine action on specific target tissues [18,19]. Among these, fibronectin type III domain-containing protein 5 (FNDC5), a transmembrane glycoprotein, is undoubtedly involved in brain and musculoskeletal adaptations to exercise [20,21]. The expression of FNDC5 and the polypeptide generated by its cleavage, irisin, is known to increase abundantly during exercise in skeletal muscle, bone, and nervous tissue [22,23,24]. Interestingly, the FNDC5/irisin action has been proposed to be expressed in brain tissue through the expression of brain-derived neurotrophic factor (BDNF), a major neurotrophin responsible for synaptic plasticity, stimulating the cognitive processes of learning and memory [25,26]. Particularly, BDNF overexpression has been proposed to correlate with increased N-methyl-D-aspartate (NMDA) receptor activity and a consequent increased neuronal calcium influx. Thus, a high BDNF content could enhance synaptic potentiation and modulate axodendritic morphology, thus positively influencing neuronal activity [27]. Such BDNF-mediated effects could be enhanced by FNDC5, as its exercise-induced upregulation is known to result in BDNF upregulation [28]. Interestingly, protective action for FNDC5 has also been suggested in neurodegenerative disorders induced by the accumulation of amyloid aggregates in the nervous system [29]. In this regard, Lourenco et al. recently found reduced FNDC5/irisin levels in the brains of mice and Alzheimer’s disease (AD) patients, in association with impaired long-term potentiation. Noteworthily, increased brain levels of FNDC5/irisin restored synaptic plasticity and prevented memory impairment, suggesting that increasing irisin levels, either pharmacologically or through exercise, is a new therapeutic strategy to counteract cognitive decline in AD [30]. In agreement, the same authors found that irisin levels in the cerebrospinal fluid of AD patients were positively correlated with increased BDNF and cognitive performance, confirming the ability of this myokine to modulate BDNF expression at the hippocampal level and providing further evidence for its involvement in neuroinflammatory responses and neurodegeneration [31].

In relation to musculoskeletal tissue, Shan et al. reported that knockout of the myostatin gene, the main negative regulator of muscle growth, produces an increase in muscle mass at the same time as browning adipose tissue, an effect known to be induced by irisin and increased FNDC5 expression [32]. Therefore, increased production of irisin through exercise could lead to increased muscle growth via a molecular pathway involving a reduction in myostatin expression [33]. Furthermore, given the stressful communication between muscle and bone tissue, the consequences of increased muscle growth, induced by exercise, could also have an impact on bone tissue, leading to an increase in the proliferative and mineralizing capacity of osteoblasts [34]. In this regard, Yang and colleagues recently observed an upregulation of osteogenic markers, such as runt-related transcription factor-2 (RUNX2), osterix, and osteopontin, in MC3T3-E1 murine osteoblasts in response to irisin, suggesting the involvement of this myokine in bone formation and mineralization [35].

Based on this evidence, in the present work, which is a continuation of previous work [9], we reproduced similar experimental conditions and enriched data previously obtained in young mice to explore the physiological adaptations to vibratory training. Specifically, the aim of our study was to (i) evaluate whether two WBV protocols, differing in vibration exposure time and recovery period between two consecutive sessions, could modulate FNDC5 expression in brain, muscle, and bone tissue of 4-month-old young mice, and (ii) investigate WBV-induced changes in the expression of tissue-specific markers, such as BDNF, myostatin, and collagen I (COL-1), to determine vibratory training-related tissue adaptations in terms of brain and musculoskeletal health.

## 2. Results

### 2.1. Modulation of Synaptic Plasticity after WBV Exposure

**Figure 1:**
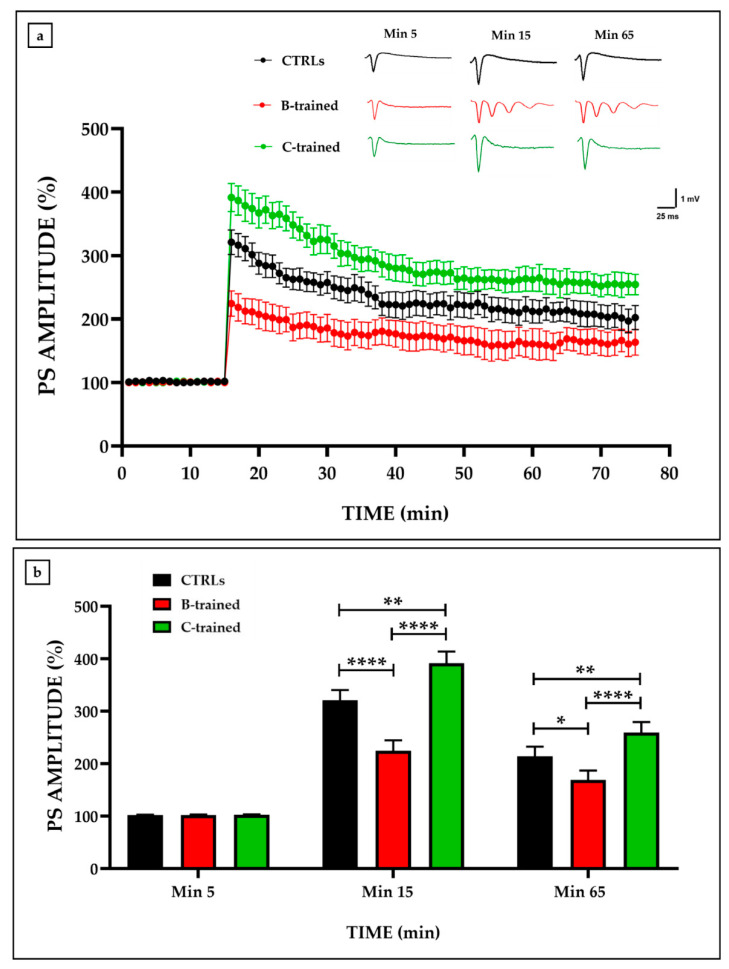
Synaptic plasticity in the CA1 hippocampal subfield of trained and sedentary mice. (**a**) Percentage population spike (PS) amplitude as a function of time after high-frequency stimulation (HFS), applied at time t = 15 (arrow), is shown in CTRLs (black line, *n* = 8 from *N* = 5), in B-trained (red line, *n* = 6 from *N* = 5), and in C-trained (green line, *n* = 7 from *N* = 5) mice slices. The insert shows representative recordings obtained from slices of each experimental group. The first curve in each group refers to the basal synaptic transmission and it was recorded prior to the HFS application, whereas the other curves represent the PS measured at 15, immediately after HF, and 65 min after HFS. (**b**) The PS amplitude values at min 5 (black bar), at min 15 (red bar), and at min 65 (green bar) from the HFS are shown for each experimental group. Bars in the plot are means ± SEM of values obtained from different slices. Note that a significant statistical difference was reported between trained and control groups at min 15 (CTRLs vs. B-trained, **** *p* < 0.0001; CTRLs vs. C-trained, ** *p* < 0.01; B-trained vs. C-trained, **** *p* < 0.0001; F = 19.92) and at min 65 (CTRLs vs. B-trained, * *p* < 0.05; CTRLs vs. C-trained, ** *p* < 0.01; B-trained vs. C-trained, **** *p* < 0.0001; F = 9.518).

In agreement with our previous data [9], the influence of vibratory training on synaptic plasticity varied depending on the protocol administered, with optimal results for the protocol characterized by a low vibration frequency and longer recovery time (Figure 1a). In fact, only the C protocol seemed to positively modulate synaptic plasticity throughout the electrophysiological recording, with population spike (PS) amplitude values after high-frequency stimulation (HFS) significantly higher than those of the other experimental groups. On the contrary, no improvement in synaptic plasticity was detected in the hippocampal slices of the B-trained group, with significantly reduced PS amplitude values, especially in the first twenty minutes after HFS. Noteworthily, the HFS application at the fifteenth min of recording also induced the onset of an epileptic trend that persisted until the end of the electrophysiological recording, as shown in the inset of Figure 1a comparing representative traces from the different experimental groups.

Figure 1b shows the PS amplitude values at min 5, before tetanic stimulation (HFS) (CTRLs: 102.0 ± 0.7; B-trained: 102.1 ± 0.8; C-trained: 102.5 ± 0.9); at min 15, immediately after HFS (CTRLs: 321.0 ± 19.3; B-trained: 224.4 ± 20.0; C-trained: 391.3 ± 22.3; CTRLs vs. B-trained, **** *p* < 0.0001; CTRLs vs. C-trained, ** *p* < 0.01; B-trained vs. C-trained, **** *p* < 0.0001; F = 19.92), and at min 65 (CTRLs: 213.9 ± 18.4; B-trained: 169.0 ± 17.7; C-trained: 259.2 ± 20.1; CTRLs vs. B-trained, * *p* < 0.05; CTRLs vs. C-trained, ** *p* < 0.01; B-trained vs. C-trained, **** *p* < 0.0001; F = 9.518).

### 2.2. Histological and Morphometric Analysis of Cerebellar and Hippocampal Tissues after WBV Exposure

**Figure 2:**
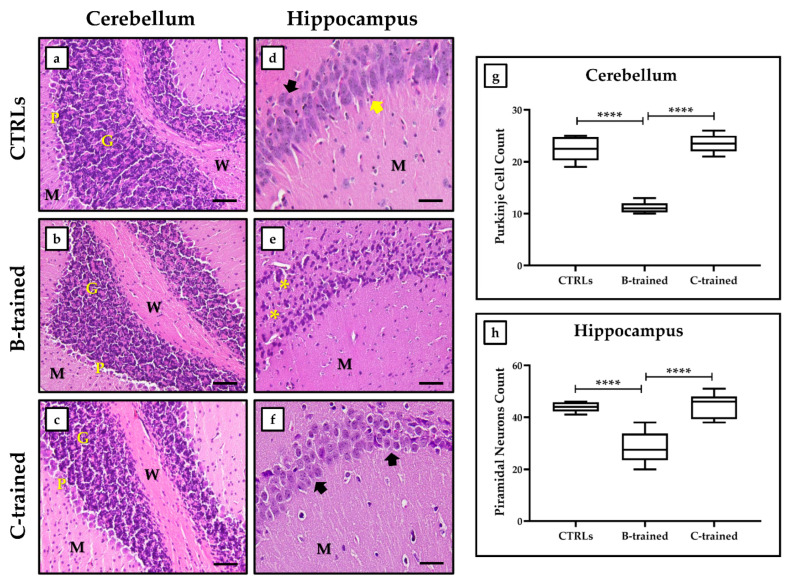
Haematoxylin and eosin (H&E)-stained sections of cerebellar and hippocampal tissues from the different experimental groups. (**a**) Cerebellar tissue of both CTRL groups: molecular cell layer with small, scattered basket cells and stellate cells; large pyriform cells in the Purkinje cell layer with open-faced vesicular nuclei, eosinophilic cytoplasm and prominent Nissl’s granules; granular cell layer with small, highly stained cells. (**b**) Cerebellar tissue of the B-trained group: smaller Purkinje cells with deeply stained nuclei and eosinophilic cytoplasm. (**c**) Cerebellar tissue of the C-trained group: larger Purkinje cells with open-faced nuclei. (**d**) Hippocampal tissue of both CTRL groups: pyramidal neurons (black arrow) of uniform size and arrangement, with rounded central vesicular nucleus and prominent nucleolus. Presence of many glial cells (yellow arrow) between the neuronal processes in the molecular layer is evident. (**e**) Hippocampal tissue of the B-trained group: reduced pyramidal neurons with hyperchromatic nuclei and some areas without neurons (asterisks). (**f**) Hippocampal tissue of the C-trained group: numerous and very crowded pyramidal neurons (black arrow), basophilic cytoplasm, well-formed Nissl’s granules, and vesicular nuclei. (**g**) The number of Purkinje cells in the cerebellar tissue is shown for each experimental group (CTRLs vs. B-trained, **** *p* < 0.0001; B-trained vs. C-trained, **** *p* < 0.0001; F = 111.0). (**h**) The number of pyramidal neurons in the hippocampal CA1 region is shown for each experimental group (CTRLs vs. B-trained, **** *p* < 0.0001; B-trained vs. C-trained, **** *p* < 0.0001; F = 32.34). M: molecular cell layer; P: Purkinje cell layer; G: granular cell layer; W: medulla formed by white matter fibers. 20× images, scale bar represents 50 μm.

As shown in Figure 2a–c, all experimental groups display a cerebellar cortex with three clearly distinguishable layers, which include the molecular cell layer (M), the Purkinje cell layer (P), and the granular cell layer (G). In addition, the medulla formed by white matter fibers (W) is also clearly visible. Notably, the cerebellar tissue of both CTRL groups (Figure 2a) showed a molecular cell layer with small, scattered basket cells and stellate cells. The Purkinje cell layer contained large pyriform cells with vesicular open-faced nuclei, eosinophilic cytoplasm, and prominent Nissl’s granules; while the granular cell layer was characterized by small, highly stained cells. On the contrary, some morphological alterations were observed in the cerebellar tissue of the B-trained group (Figure 2b). Indeed, Purkinje cells were smaller with respect to the other experimental groups, with deeply stained nuclei and eosinophilic cytoplasm. Noteworthily, the C-trained group (Figure 2c) showed normal architecture of the three cortical layers of the cerebellum, with particular attention to the Purkinje cell layer in which cells with open-faced nuclei had increased in size when compared to the B-trained group.

Relative to the hematoxylin and eosin (H&E) sections of the hippocampus, both CTRL groups (Figure 2d) showed a regular architecture of the CA1 region characterized by a cell layer of uniformly sized and arranged pyramidal neurons. In addition, each neuron had a rounded central vesicular nucleus with a prominent nucleolus, while the molecular layer contained many glial cells between the neuronal processes. H&E-stained sections of the hippocampus of the B-trained group (Figure 2e) showed markedly affected pyramidal cells of the CA1 region, since most of the pyramidal neurons were shrunken with hyperchromatic nuclei. In addition, areas without pyramidal neurons were observed. In contrast, histological analysis of the hippocampal tissue of the C-trained group (Figure 2f) showed a normal architecture of the CA1 region, consisting of very crowded cells with basophilic cytoplasm, well-formed Nissl’s granules, and vesicular nuclei.

In agreement with the histological evaluation, morphometric analysis showed significant variations in both the number of Purkinje cells in the cerebellum and the number of hippocampal pyramidal neurons between the various experimental groups. Specifically, the number of Purkinje cells (Figure 2g) was 22.4 ± 0.8 in the CTRL groups, 11.3 ± 0.4 in the B-trained group, and 23.5 ± 0.6 in the C-trained group (CTRLs vs. B-trained, **** *p* < 0.0001; B-trained vs. C-trained, **** *p* < 0.0001; F = 111.0); while the number of hippocampal pyramidal neurons (Figure 2h) was 43.9 ± 0.6 in the CTRL groups, 28.3 ± 1.7 in the B-trained group, and 44.5 ± 1.7 in the C-trained group (CTRLs vs. B-trained, **** *p* < 0.0001; B-trained vs. C-trained, **** *p* < 0.0001; F = 32.34).

### 2.3. WBV-Induced Changes in BDNF and FNDC5 Expression in Cerebellar and Hippocampal Tissues

**Figure 3:**
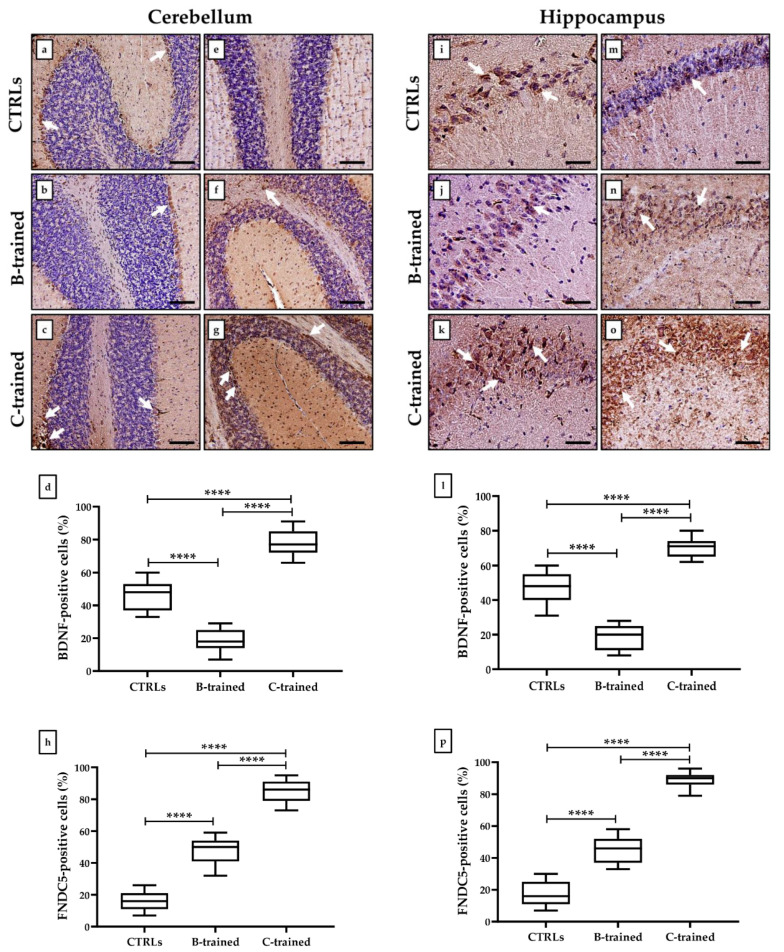
Brain-derived neurotrophic factor (BDNF) and fibronectin type III domain-containing protein 5 (FNDC5) expression analysis in cerebellar and hippocampal tissues of trained and sedentary mice by immunohistochemistry. (**a**–**c**) Arrows indicate representative BDNF-positive cells in the cerebellum. (**d**) The highest number of BDNF-positive cells was found in the cerebellum of the C-trained group (**** *p* < 0.0001; F = 209.7). (**e**–**g**) Arrows indicate representative FNDC5-positive cells in the cerebellum. (**h**) The highest number of FNDC5-positive cells was found in the cerebellum of the C-trained group (**** *p* < 0.0001; F = 353.2). (**i**–**k**) Arrows indicate representative BDNF-positive cells in the hippocampus. (**l**) The highest number of BDNF-positive cells was found in the hippocampus of the C-trained group (**** *p* < 0.0001; F = 198.1). (**m**–**o**) Arrows indicate representative FNDC5-positive cells in the hippocampus. (**p**) The highest number of FNDC5-positive cells was found in the hippocampus of the C-trained group (**** *p* < 0.0001; F = 413.3). 20× images, scale bar represents 50 μm.

Cells positive for BDNF and FNDC5 in the cerebellar and hippocampal tissues were detected by immunohistochemical analysis and expressed as a percentage of the total analyzed. As shown in Figure 3, a similar pattern of BDNF expression was detected in both the cerebellum (Figure 3a–c) and the hippocampus (Figure 3i–k), although with differences between groups. In fact, the highest expression of BDNF was observed in the C-trained group, since the relative number of BDNF-positive cells was significantly increased compared to the other experimental groups. In contrast, BDNF expression was significantly reduced in the cerebellar and hippocampal tissues of animals trained with the B protocol. In fact, the average percentage of BDNF-positive cells in the cerebellum was 46.3 ± 2.3 in the CTRL groups, 18.7 ± 1.8 in the B-trained group, and 78.5 ± 2.1 in the C-trained group (**** *p* < 0.0001; F = 209.7) (Figure 3d). Similarly, the average percentage of BDNF-positive cells in the hippocampus was 47.7 ± 2.3 in the CTRL groups, 18.6 ± 1.8 in the B-trained group, and 70.2 ± 1.3 in the C-trained group (**** *p* < 0.0001; F = 198.1) (Figure 3l).

In agreement, FNDC5 also showed a similar expression pattern in both the cerebellum (Figure 3e–g) and the hippocampus (Figure 3m–o). Indeed, both the cerebellum and hippocampus of the C-trained group had the highest expression of FNDC5, with a significantly higher relative number of FNDC5-positive cells compared to both CTRL and B-trained groups. Interestingly, a significant increase in the relative number of FNDC5-positive cells was also found in the B-trained group compared to the sedentary animals. In fact, the average percentage of FNDC5-positive cells in the cerebellum was 16.2 ± 1.5 in the CTRL groups, 47.5 ± 2.2 in the B-trained group, and 85.1 ± 1.8 in the C-trained group (**** *p* < 0.0001; F = 353.2) (Figure 3h). Likewise, the average percentage of FNDC5-positive cells in the hippocampus was 17.2 ± 1.8 in the CTRL groups, 45.5 ± 2.2 in the B-trained group, and 89.3 ± 1.2 in the C-trained group (**** *p* < 0.0001; F = 413.3) (Figure 3p).

### 2.4. Histological and Morphometric Analysis of Muscle and Bone Tissues after WBV Exposure

**Figure 4:**
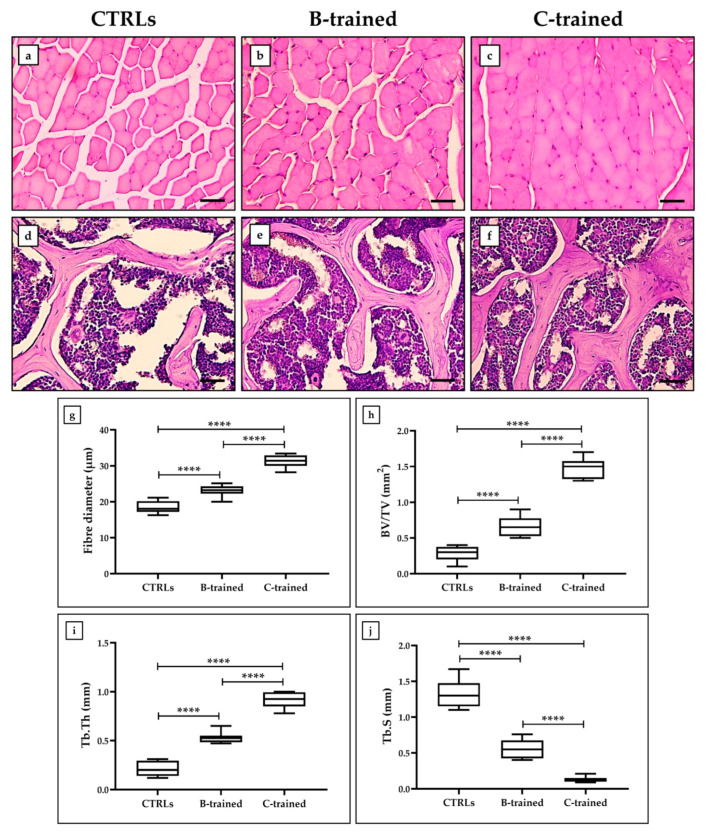
Hematoxylin and eosin (H&E)-stained sections of muscle and bone tissues from the different experimental groups. (**a**–**c**) Polygonal and multinucleated skeletal muscle fibers and peripheral nuclei were evident in the muscle tissue of each experimental group. A complete organization of the fibers into fascicles was observed in the C-trained group. (**g**) The muscle fiber diameter is shown for each experimental group (**** *p* < 0.0001; F = 114.8). (**d**–**f**) Thicker and more voluminous trabecular bones were observed in the C-trained group. (**h**) The bone volume (BV/TV) is shown for each experimental group (**** *p* < 0.0001; F = 180.7). (**i**) The trabecular thickness (Tb.Th) is shown for each experimental group (**** *p* < 0.0001; F = 189.4). (**j**) The trabecular separations (Tb.S) is shown for each experimental group (**** *p* < 0.0001; F = 160.4). For 20× images, scale bar represents 50 μm.

Histological analysis of the muscle tissue showed a normal tissue architecture, characterized by skeletal muscle fibers of polygonal shape, multinucleated with peripheral nuclei (Figure 4a–c). Particularly, a complete organization of the fibers into fascicles was evidenced in the C-trained group. However, morphometric analysis (Figure 4g) showed significantly lower values of the muscle fiber diameter in the CTRL groups (18.5 ± 0.6) than in the two trained groups (B-trained: 23.1 ± 0.6; C-trained: 31.2 ± 0.6; **** *p* < 0.0001; F = 114.8). Interestingly, the mice trained with the C protocol showed the highest muscle fiber diameter values.

Characteristic histological findings in trabecular bone were observed in each experimental group (Figure 4d–f) and confirmed by the analysis of conventional bone morphometric parameters, such as bone volume (BV/TV), trabecular thickness (Tb.Th), and trabecular separations (Tb.S). Noteworthily, the C-trained group had greater bone volume and trabecular thickness (Figure 4h,i). In fact, BV/TV values were 0.3 ± 0.03 for the CTRL groups, 0.7 ± 0.05 for the B-trained group and 1.5 ± 0.05 for the C-trained group (**** *p* < 0.0001; F = 180.7). Similarly, Tb.Th values were 0.2 ± 0.03 for the CTRL groups, 0.5 ± 0.02 for the B-trained group and 0.9 ± 0.03 for the C-trained group (**** *p* < 0.0001; F = 189.4). In contrast, a greater separation of the trabeculae was observed in sedentary animals (Figure 4j), with Tb.S values of 1.3 ± 0.1 for the CTRL groups, 0.6 ± 0.1 for the B-trained group and 0.1 ± 0.1 for the C-trained group (**** *p* < 0.0001; F = 160.4).

### 2.5. WBV-Induced Changes in Myostatin, COL-1, and FNDC5 Expression in Muscle and Bone Tissues

**Figure 5:**
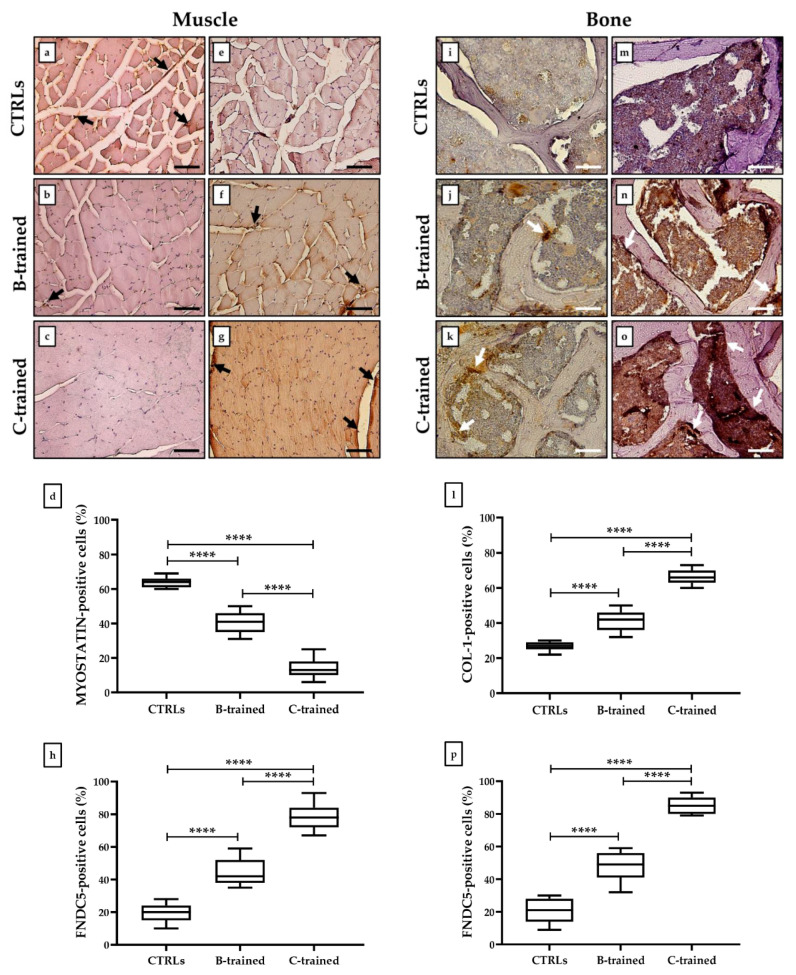
Myostatin, collagen I (COL-1) and fibronectin type III domain-containing protein 5 (FNDC5) expression analysis in muscle and bone tissues of trained and sedentary mice by immunohistochemistry. (**a**–**c**) Black arrows indicate representative myostatin-positive cells in the muscle tissue. (**d**) The highest number of myostatin-positive cells was found in the muscle of the CTRLs group (**** *p* < 0.0001; F = 334.9). (**e**–**g**) Black arrows indicate representative FNDC5-positive cells in the muscle tissue. (**h**) The highest number of FNDC5-positive cells was found in the muscle of the C-trained group (**** *p* < 0.0001; F = 249.3). (**i**–**k**) White arrows indicate representative COL-1-positive cells in the bone tissue. (**l**) The highest number of COL-1-positive cells was found in the bone of the C-trained group (**** *p* < 0.0001; F = 354.9). (**m**–**o**) White arrows indicate representative FNDC5-positive cells in the bone tissue. (**p**) The highest number of FNDC5-positive cells was found in the bone of the C-trained group (**** *p* < 0.0001; F = 307.0). For 20× images, scale bar represents 50 μm.

Cells positive for myostatin, COL-1, and FNDC5 in the muscle and bone tissues were detected by immunohistochemical analysis and expressed as a percentage of the total analyzed. Figure 5 shows different expression patterns for myostatin and COL-1, depending on the experimental group. Indeed, the highest expression of myostatin was detected in the muscle tissue of sedentary animals, as the relative number of myostatin-positive cells was significantly increased compared to the other experimental groups. In contrast, myostatin expression was significantly reduced in the muscle tissue of the trained group (Figure 5a–d) (CTRLs group: 65.7 ± 1.1; B-trained: 40.5 ± 1.6; C-trained: 14.1 ± 1.5; **** *p* < 0.0001; F = 334.9). Relative to the bone tissue, the C-trained group showed the highest expression of COL-1, whereas its levels were significantly reduced in the other experimental groups (Figure 5i–l) (CTRLs group: 26.8 ± 0.7; B-trained: 41.4 ± 1.3; C-trained: 66.2 ± 1.1; **** *p* < 0.0001; F = 354.9).

FNDC5 showed a similar expression pattern in both muscle (Figure 5e–g) and bone (Figure 5m–o). Notably, both tissues taken from the C-trained group showed the highest expression of FNDC5, with a significantly higher relative number of FNDC5-positive cells compared to the CTRL and B-trained groups. Furthermore, a significant increase in the relative number of FNDC5-positive cells was also found in the B-trained group compared to the sedentary animals. In fact, the average percentage of FNDC5-positive cells in the muscle was 19.5 ± 1.4 in the CTRL groups, 45.0 ± 2.1 in the B-trained group, and 78.8 ± 2.01 in the C-trained group (**** *p* < 0.0001; F = 249.3) (Figure 5h). Similarly, the average percentage of FNDC5-positive cells in the bone was 20.5 ± 1.9 in the CTRL groups, 47.9 ± 2.2 in the B-trained group, and 85.6 ± 1.3 in the C-trained group (**** *p* < 0.0001; F = 307.0) (Figure 5p).

## 3. Discussion

Our previous studies have shown that vibratory training is an effective strategy to improve brain health and cognitive functions, as well as to counteract muscle atrophy and motor decline related to aging and/or a sedentary lifestyle [9,16]. In agreement, numerous studies over the last decades report among the main benefits of mechanical vibrations those affecting the musculoskeletal system, such as increased bone mass and muscle strength, as well as increased hormonal responses and improved cognitive abilities, anxious and depressive behavior, and neuromuscular adaptation [36,37]. However, cellular and molecular mechanisms through which vibratory training exerts its effects, influencing overall health, are largely unknown. Understanding the biological processes responsible for physiological adaptations to WBV is a key step in the development of appropriate vibratory training protocols tailored to personal needs. Indeed, WBV could be used as a form of passive training for patients unable to exercise, to prevent and/or counteract the cognitive and motor decline associated with sedentariness and the progression of chronic degenerative diseases [38]. To the best of our knowledge, this is the first experimental study to determine whether exposure to WBV for 3 months can modulate the FNDC5 expression, together with that of tissue-specific markers such as BDNF, myostatin, and COL-1 in the brain, muscle, and bone tissue, respectively.

### 3.1. WBV Effects on the Brain System

In agreement with our previous work [9], an increase in PS amplitude values was detected in the hippocampal slices of the C-trained group, showing that this protocol exerts a positive modulation of synaptic plasticity through the stimulation of cognitive processes of learning and memory. In contrast, mice trained with the B protocol showed a marked reduction in synaptic plasticity compared to sedentary animals, concomitant with the epileptic tendency development, suggesting the onset of structural brain damage. In this regard, histological and morphometric analysis performed on cerebellar and hippocampal tissues of C-trained mice showed complete preservation of structural organization, confirming the findings of gain of synaptic function. In contrast, brain tissue from the B-trained group revealed the presence of structural alterations, such as reduced Purkinje cells in the cerebellum and shrunken or even absent pyramidal neurons in the hippocampus.

Immunohistochemical analysis performed on the cerebellum and hippocampus showed a higher number of FNDC5-positive cells in the tissues of protocol C-trained mice than in the remaining experimental groups. However, we surprisingly observed that tissues from the B-trained group also showed higher expression of FNDC5 compared to the sedentary mice, indicating that the cerebellar and hippocampal structural changes were not dependent on the absence of this multifunctional protein. Therefore, we performed immunohistochemical analysis for BDNF and found a markedly altered expression pattern, depending on the WBV protocol used. Indeed, while the brain tissue of the C-trained group was characterized by a significant increase in BDNF-positive cells compared to the sedentary animals, the tissues of mice trained with the B protocol had very few cells positive for neurotrophin, confirming its role in the health of the nervous system and brain function.

Physical exercise is already known to promote cognitive function through the BDNF expression [39]. Noteworthily, FNDC5 has been proposed as one of the possible responses for this effect, through peroxisome proliferator-activated receptor gamma coactivator 1-alpha (PGC-1α)-mediated regulation, a highly conserved co-activator of transcription factors that preserves and protects neurons from destruction. In this regard, Wrann et al. observed that PGC-1α-induced increased expression of FNDC5 in murine primary cortical and hippocampal neurons promotes BDNF expression [26]. Furthermore, the PGC-1α/FNDC5/BDNF pathway has been suggested to activate positive feedback on the activity of the cyclic AMP response element binding protein (CREB), leading to the PGC-1α expression enhancement. In this context, FNDC5 assumes a role as a key regulator of the effects of exercise on brain function and health by regulating BDNF production [40]. Finally, FNDC5 has also been proposed as a regulator of hippocampal neurogenesis, as more hippocampal neurons were detected after irisin administration in the murine H19-7 HN cell line [41].

Our results agree with scientific evidence in the literature, as they show that movement, understood as vibratory training, increases FNDC5 production, in association with improved structural organization of tissue and increased BDNF expression. Surprisingly, the brain tissue of mice trained with the B protocol showed an increase in FNDC5 expression, concomitantly with cerebellar and hippocampal alterations and a reduced presence of BDNF-positive cells. Overall, molecular mechanisms responsible for the physiological adaptations to vibration have not yet been fully elucidated, as additional regulators of neurogenesis and neuronal plasticity may be involved in the brain response to vibration and result in dramatically different effects depending on the vibratory training protocol used.

### 3.2. WBV Effects on the Musculoskeletal System

Morphometric analysis by light microscopy showed that the mean diameter of the muscle fibers of the sedentary animals was significantly lower than that of the vibration-trained mice, in association with an abundant amount of interfibrillar connective tissue. Interestingly, among the groups of trained mice, those exposed to the C training protocol had the largest mean diameter of muscle fibers and the least amount of interfibrillar connective tissue, confirming results previously obtained on 12-month-old mice [16]. Similar data were observed on bone tissue, as the sedentary animals had less bone volume and trabecular thickness and greater trabecular separation than the trained mice. Noteworthily, again the bone tissue of the C-trained group showed the best qualitative characteristics, such as higher bone volume and trabecular thickness, at the same time as lower trabecular separation.

Immunohistochemical analysis showed increased expression of FNDC5 in both muscle and bone of mice undergoing vibratory training compared to sedentary animals. Particularly, the C-trained group showed the highest number of FNDC5-positive cells, again highlighting the beneficial potential of this protocol on the musculoskeletal system. The structural improvements induced by the different WBV protocols were confirmed by immunohistochemical analysis for two tissue-specific markers, myostatin in muscle and COL-1 in bone. As expected, muscle sections from sedentary mice showed the greatest presence of myostatin-positive fibers, while muscles from trained mice, and specifically the C-trained group, were weakly positive for myokine, suggesting vibratory training as a good strategy to preserve muscle mass. Similarly, bone tissue from C-trained mice showed increased expression of COL-1, which was significantly lower, but not absent, in sedentary mice.

Overall, our results suggest that vibratory training exerts a powerful beneficial effect on musculoskeletal health by improving muscle and bone mass and regulating the synthesis of tissue-specific markers. To the best of our knowledge, there are no studies investigating the relationship between FNDC5 and myostatin because of WBV. However, Shan and colleagues demonstrated that knockout of the myostatin gene upregulates FNDC5 production in muscle through increased PGC-1α [32]. Furthermore, myostatin inhibition and exercise are known to increase both mRNA and protein levels of PGC-1α, suggesting the FNDC5 involvement in the muscle mass increase induced by the absence of myostatin [42,43]. Interestingly, an anti-osteogenic role of myostatin has also been proposed, as mice deficient in this protein are characterized by an expansion of muscle insertion sites in the humerus, femur, and spine, associated with an increase in trabecular area and humeral bone mineral content [44]. Furthermore, FNDC5 is known to contribute to bone metabolism by promoting the proliferation and differentiation of osteoblasts and reducing the number of osteoclasts [45,46]. Therefore, a properly designed vibratory training protocol could promote musculoskeletal well-being through the FNDC5 production and by regulating the expression of key markers, such as myostatin, with effects on both muscle and bone tissue.

## 4. Materials and Methods

### 4.1. Animals

Twenty 4-month-old male mice of the wild-type BALB/c strain were used, following the procedures established by the European Union Council Directive 2010/63/EU for animal experiments [47]. All experimental protocols were approved by the Italian Ministry of Public Health (authorization no. 86/2018-PR).

The animals were divided into four groups (five mice per group): two groups of trained mice, each subjected to a specific WBV protocol, and two control groups. Specifically, we used a control group (CTRL SED) with sedentary mice not subjected to any type of training, and another control group (CTRL WBV) with mice subjected to the same regime of placement on the box in the platform, the same environmental exposure including motor sounds, but not exposed to vibratory training. Each evaluation was carried out on both control groups. However, due to the almost complete overlap of the results and to avoid redundancy, representative data and images of the CTRL WBV were shown for each measurement performed.

All experimental animals were kept under the same housing and dietary conditions and underwent daily checks of their physical condition, including coat and skin condition, weight, and body functions by resident and experimental veterinarians.

### 4.2. Vibratory Training Protocols

Vibration training sessions were performed using a vibrating platform (Power Club, Vigarano Mainarda, 44,049 FE, Italy). As previously described [16], it is characterized by a power supply of 220 V, a total maximum electrical power of 0.12 KW, a minimum vibration frequency of 45 Hz, and a maximum vibration frequency of 90 Hz. The minimum vibration frequency results in an acceleration of 2 g and a shift of 1.5 mm, while the maximum vibration frequency results in an acceleration of 2.8 g and a shift of 1.1 mm.

Animals were subjected to two types of WBV protocol, equal in vibration frequency (45 Hz), but different in vibration exposure time and recovery period between two successive series. Specifically, one WBV protocol (B-trained) was characterized by 5 vibration series of 3 min each with 1 min of recovery, while the other WBV protocol (C-trained) consisted of 3 vibration series of 2 min and 30 s each with 2 min and 30 s of recovery. Vibratory training was spread over 12 weeks, with a frequency of 3 weeks, for a total of 36 training sessions. The animals were raised on a light: dark cycle of 12:12 h, and training was carried out in the morning, between 10 and 11 a.m.

### 4.3. Synaptic Plasticity by Extracellular Recordings in Mouse Hippocampal Slices

All animals, sedentary and trained, under anesthesia with halothane (2-Brom-2-chlor-1,1,1-trifuor-ethan) were sacrificed at the end of the experimental period, minimizing their suffering. Their brains were quickly removed and placed in cold, oxygenated artificial cerebral spinal fluid (ACSF) containing the following (in mM): NaCl, 124; KCl, 2; KH_2_PO_4_, 1.25; MgSO_4_, 2; CaCl_2_, 2; NaHCO_3_, 26; and glucose, 10. The hippocampus was rapidly dissected and cut transversely into 450 μm thick slices using a McIlwain tissue chopper (Mickle Laboratory Engineering Co., Gomshall, UK). Then, hippocampal slices were transferred to a tissue chamber, where they were laid in an interface between oxygenated ACSF and humidified gas (95% O_2_, 5% CO_2_) at 32–34 °C (pH = 7.4), constantly superfused at a flow rate of 1.2 mL/min.

Extracellular recordings of the PSs were made in the stratum pyramidale of the CA1 subfield using glass microelectrodes filled with 2M NaCl (resistance 5–10 MW). Orthodromic stimuli (10–500 mA, 20–90 ms, 0.1 Hz) were delivered through a platinum electrode placed in the Schaffer collaterals in the stratum radiatum. The test stimulus intensity of 50 ms square pulses was adjusted to provide a PS of 2–4 mV at 0.03 Hz. Each minute, a trace was calculated as the average of six recordings every 10 s. A high-frequency stimulation (HFS, 100 Hz, 1 s), after the recording of stable signals (15–20 min), was given to assess changes in PS amplitude, which was expressed as a percentage of the basal PS amplitude. Signals were fed to an Axoclamp-2A amplifier (Foster City, CA, USA), acquired through a digital/analogic system (Digidata 1440A, Axon Instruments, Foster City, CA, USA), and analyzed with pCLAMP10 software (Axon Instruments, Foster City, CA, USA).

### 4.4. Histological and Morphometric Analysis

In addition to the electrophysiological evaluation, a histological and morphometric analysis was conducted by collecting tissue samples from cerebellum, hippocampus, muscle, and bone from the same animals immediately after sacrifice. These were immediately fixed in 4% paraformaldehyde for 24 h and embedded in paraffin. Then,3 μm thick sections were stained with hematoxylin and eosin (H&E) (Bio-Optica, Milan, Italy) to perform morphological analysis. H&E slides were visualized by a Nikon upright microscope ECLIPSE Ci-S (Nikon Corporation, Tokyo, Japan) connected to a Nikon digital camera. Images were acquired at 20× magnification using NIS-Elements software (5.30.01; Laboratory Imaging, Prague, Czech Republic). For morphometric analysis, two blinded observers counted Purkinje cells in the cerebellum and pyramidal neurons in the CA1 region of the hippocampus by taking a total of 8 non-overlapping readings for each experimental animal. Similarly, the evaluation of muscle fiber diameter and bone morphometric parameters was conducted. In all cases, measurements were performed at higher magnification (40×, scale bar 25 µm) In addition, the reference area used for the cell count was set using the NIS-Elements software, so the size of the region of interest was the same at each cell count.

### 4.5. Immunohistochemistry

BDNF, myostatin, COL-1, and FNDC5 expressions were assessed in the experimental samples by immunohistochemical analysis. Briefly, 3 μm-thick sections were pretreated with EDTA citrate (pH 6.0) for 20 min at 95 °C and then incubated for 1 h with mouse monoclonal anti-BDNF [3B2] (clone ab205067, AbCam, Cambridge, United Kingdom), rabbit polyclonal anti-myostatin propeptide (clone ab134682, AbCam, Cambridge, United Kingdom), mouse monoclonal anti-COL-1 (GTX26308, GeneTex, North America), and rabbit polyclonal anti-FNDC5 C-terminal (clone ab181884, AbCam, Cambridge, United Kingdom). Washings were performed with PBS/Tween20 (pH 7.6) (UCS Diagnostic, Rome, Italy); horseradish peroxidase (HRP)-3,3′ diaminobenzidine (DAB) Detection Kit (UCS Diagnostic, Rome, Italy) was used to reveal immunohistochemical reactions. Specifically, 50 μL DAB/450 μL of substrate was incubated for 3 min. To assess the background of immunostaining, we included negative controls for each reaction (Figures S1 and S2) by incubating the sections with secondary antibodies (HRP) alone or a detection system (DAB) alone.

Immunopositive cells for BDNF, myostatin, COL-1 and FNDC5 were detected using NIS-Elements software (5.30.01; Laboratory Imaging, Prague, Czech Republic) and expressed as a percentage of the total analyzed for BDNF, myostatin, COL-1 and FNDC5. For each condition, the experiment was conducted in triplicate (*n* = 15 from *N* = 5 experiments).

### 4.6. Western Blotting

A western blotting analysis was performed to distinguish the different isoforms of BDNF and ensure that the observed changes in BDNF levels depended on the mature form, which has a molecular weight of approximately 14 kDa (Figure S1). Cell proteins extracted by using RIPA buffer were separated by 8–16% precast SDS-PAGE (Bio-Rad, Hercules, CA, USA) under reduced conditions. Protein concentration was determined using the Pierce BCA Protein Assay Kit (Thermo Scientific, Vilnius, Lithuania). Equal amounts of protein (15 μg) were resolved on 10% SDS-PAGE and transferred to PVDF membrane. Then membranes were incubated with a mouse monoclonal anti-BDNF [3B2] (clone ab205067, AbCam, Cambridge, United Kingdom; 1:1000) and successively with anti-mouse IgG coupled to HRP. Immunoreactive electrophoretic bands were detected by enhanced chemiluminescence (ECL Advance, Amersham; GE Healthcare Life Sciences, Little Chalfont, Buckinghamshire, UK) using a VersaDoc 5000 Imager (Bio-Rad).

### 4.7. Statistical Analysis

All statistical analyses were performed using GraphPad Prism 8 software (GraphPad Prism 8.0.1, La Jolla, CA, USA). For electrophysiological experiments, data were expressed as the mean ± SEM, with *n* representing the number of slices analyzed and *N* indicating the number of mice used. Data were compared with one-way ANOVA and Tukey’s multiple comparison test and were considered significantly different if *p* < 0.05. For morphometric and immunohistochemical analysis, data were expressed as mean ± SEM, and *n* represents the percentage of BDNF, myostatin, COL-1, and FNDC5 positive cell number. Data were compared with one-way ANOVA and Tukey’s multiple comparison test and were considered significantly different if *p* < 0.05.

## 5. Conclusions

The physiological adaptations to WBV are extremely variable and depend on the vibratory training protocol used. The in-depth study of the molecular mechanisms involved in the vibration response is crucial for developing vibratory training protocols that are appropriately designed based on personal needs and maximize their positive effects. Furthermore, our results suggest that the brain system is particularly susceptible to vibration and the development and application of a WBV protocol cannot disregard this. Therefore, it is necessary both to investigate the effect of vibratory training on additional organs and tissues to determine their sensitivity and to further explore the role of potential molecular actors involved in the physiological response to vibration.

## Data Availability

The data presented in this study are available on request from the corresponding author.

## Supplementary Materials

The following supporting information can be downloaded at: https://www.mdpi.com/article/10.3390/ijms231810388/s1.
