# Supplementary material for: Whole Body Vibration Improves Brain and Musculoskeletal Health by Modulating the Expression of Tissue-Specific Markers: FNDC5 as a Key Regulator of Vibration Adaptations
Source: Int J Mol Sci. 2022 Sep 8;23(18):10388. doi: 10.3390/ijms231810388 (PMC9498983; doi:10.3390/ijms231810388)
Supplement: Supplementary file 1 [file ijms-23-10388-s001.zip › ijms-1881210-supplementary.pdf]

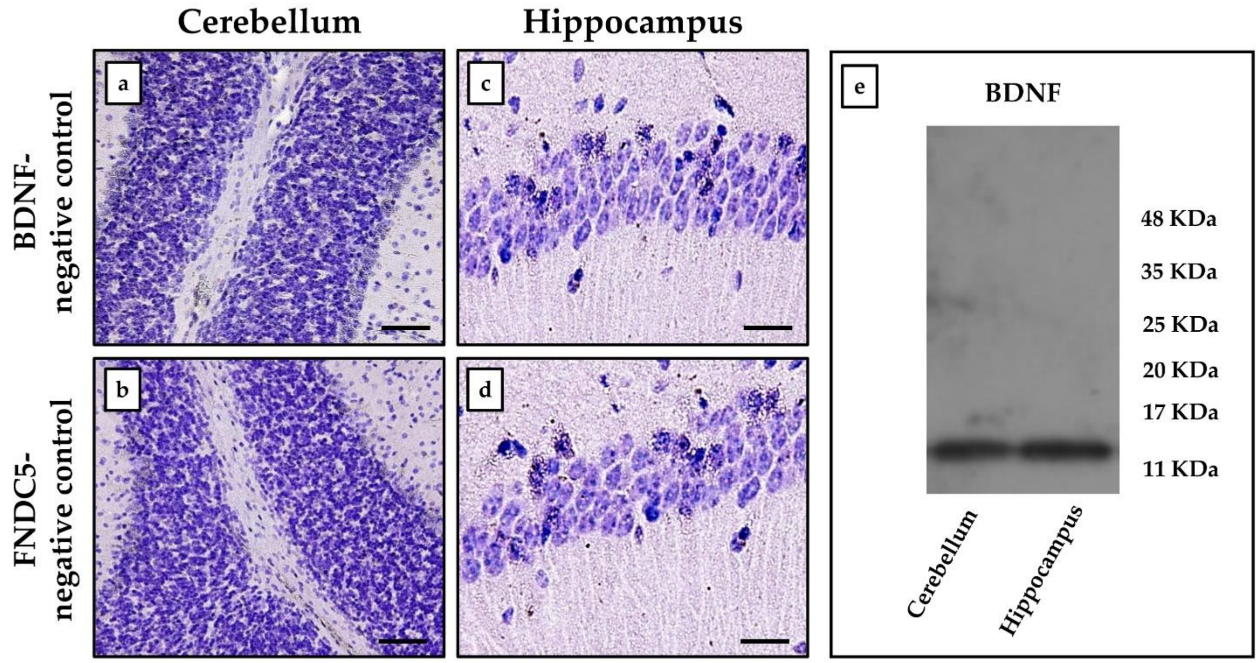

Figure S1. Immunohistochemistry and western blotting analysis in brain tissues. (**a**) BDNF-negative control in cerebellar tissue. (**b**) FNDC5-negative control in cerebellar tissue. (**c**) BDNF-negative control in hippocampal tissue. (**d**) FNDC5-negative control in hippocampal tissue. In all panels, 20× images, scale bar represents 50 μm. (**e**) Western blotting analysis on cerebellar and hippocampal tissues to distinguish BDNF isoforms. The band shown corresponds to the mature form of BDNF, with a molecular weight of approximately 14 kDa.

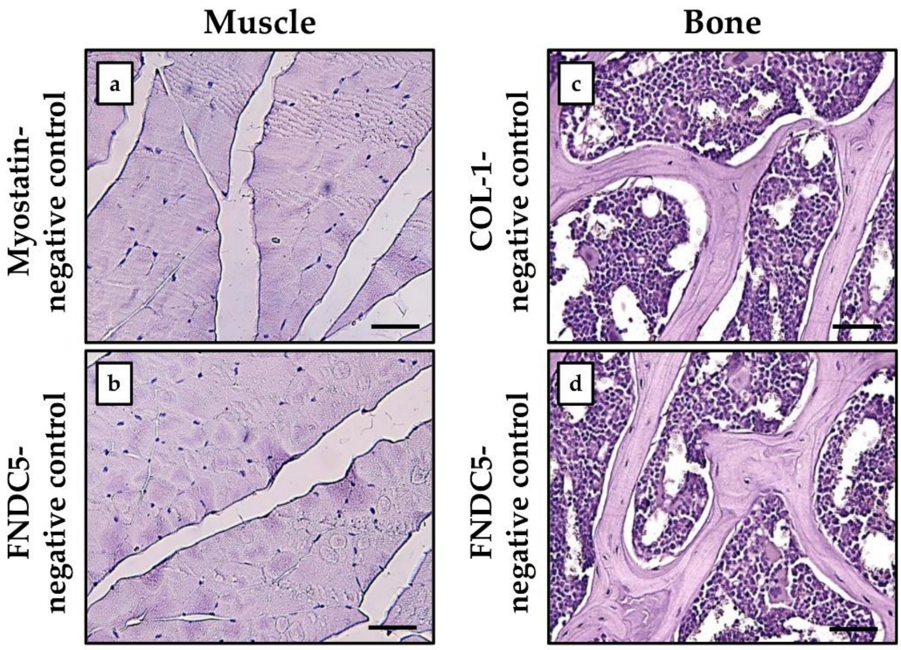

Figure S2. Immunohistochemistry analysis in musculoskeletal tissues. (**a**) Myostatin-negative control in muscle tissue. (**b**) FNDC5-negative control in muscle tissue. (**c**) COL-1-negative control in bone tissue. (**d**) FNDC5-negative control in bone tissue. 20× images, scale bar represents 50 μm.
